# Comparing Job Satisfaction Among Healthcare Workers at Emergency Departments and Primary Healthcare Units During the COVID-19 Pandemic

**DOI:** 10.7759/cureus.44974

**Published:** 2023-09-10

**Authors:** Abdulmalik Aloriney, Norah A Almudawi, Fay K Almudairs, Khawlah S Aldehailan, Murdhi H AlAnazi, Reenad F Almohaish, Lorina Badger-Emeka

**Affiliations:** 1 Department of Family Medicine/Diabetology, College of Medicine, Imam Mohammad Ibn Saud Islamic University, Riyadh, SAU; 2 Department of Medicine, Imam Mohammad Ibn Saud Islamic University, Riyadh, SAU; 3 Department of Medicine, King Faisal University, Al-Ahsa, SAU; 4 Emergency Medical Services, Prince Mohammed Bin Abdulaziz Hospital, Riyadh, SAU; 5 Medical Microbiology Division, Department of Biomedical Sciences, College of Medicine, King Faisal University, Al-Ahsa, SAU

**Keywords:** primary healthcare center, emergency department, healthcare workers, covid-19, job satisfaction

## Abstract

Background

The primary healthcare professionals' work description changed during the COVID-19 pandemic, as was the case of all other healthcare departmental operation systems.

Objectives

This investigation compares job satisfaction between emergency department (ED) and primary healthcare professionals during the COVID-19 pandemic to ascertain the possible effect of the pandemic on healthcare providers.

Methods

A cross-sectional online self-assessment questionnaire consisting of 36 questions was distributed using available social media to target all frontline healthcare workers (HCWs) in emergency departments and primary healthcare centres in Riyadh. The inclusive criterion was that the respondents should have been frontline HCWs during the pandemic era. The questionnaire was validated by a pre-test of responses of 10 frontline HCWs. This was to ensure the comprehensibility and validity of the questions. Thereafter, necessary corrections were made to the final questionnaire. Responses were collected with an Excel sheet (Microsoft Corp., Redmond, WA), while data were analysed with SPSS version 23 (IBM Corp., Armonk, NY) and GraphPad Prism version 9.2.0 (GraphPad Software, San Diego, CA)*.*

Results

The targeted sample size was 400; however, 159 HCWs responded to the questionnaire and were thus included in the investigation. There were more male (60.4%) than female (39.6%) respondents, the majority of whom were Saudi nationals (86.6%) while the remaining were non-Saudi nationals working in the Kingdom. Also, 67% of the respondents were emergency medical service professionals while the remaining (23%) were primary healthcare professionals. Significantly, 71.8% of the respondents (p < 0.05) disagreed with adequate enumeration, rewards, and chances of promotion compared to those who agreed (28.2%) during the COVID-19 pandemic. Job satisfaction was not significantly correlated to gender or the work departments (p > 0.05). Respondents significantly (p < 0.05) agreed to the competence of their supervisors, and liked their colleagues and work environment.

Conclusion

The study has shown that although supervision during the pandemic era was with competence; however, hours of work put in by these frontline emergency professionals were not adequately remunerated. Also, the services they provide seemed not to have been appreciated and hence did not lead to promotion either. Therefore, there was job satisfaction. As expected, the workload was huge while chances of promotion were lacking. These observations could lead to a substandard service should there be another pandemic. There is a need for all stakeholders to look into this more cautiously should there be another pandemic.

## Introduction

Healthcare workers (HCWs) in emergency departments have to deal with life-threatening conditions daily. The high patient burden coupled with long hours of work, shift rotations, and endless demands on physicians exposes HCWs to both physical and emotional challenges [1]. On the other hand, the role of primary healthcare centres (PHCs) differs from that of emergency medical services (EMS) in that PHCs provide health care for the community as the first point of contact [2]. With the first case of COVID-19 reported in Saudi Arabia in early 2020 [3], the responsibilities of PHC professionals as well as other healthcare departments changed [4]. COVID-19, which is caused by the new severe acute respiratory syndrome coronavirus 2 (SARS-CoV-2), spread from China rapidly to other regions of the world, putting more demands on healthcare facilities globally [5,6]. With the sudden and rapid spread of COVID-19, most of the medical staff had to join the frontlines where they participated in inspection, screening, specimen collection, and other responsibilities [7]. In the battle against COVID-19, medical teams from all healthcare departments were assigned the overall task of saving patients' lives while also preventing and controlling the pandemic's spread. Thus, HCWs were faced with an enormous workload [8,9], a huge physical burden [10], mental stress [11,12], and various other factors that challenged job satisfaction [13].

Job satisfaction is defined as a compilation of feelings, emotional responses, and beliefs that define the degree to which workers like their jobs [14]. The American Psychological Association defines job satisfaction as "the attitude of a worker towards his or her job, often expressed as a sensual response of liking or disliking the work itself, the rewards (pay, promotions, recognition), or the context (working conditions, colleagues)" [15]. Job satisfaction is associated with factors that could be personal as well as job-related [16,17]. Also, the COVID-19 pandemic was a contributory factor to HCWs' satisfaction at their jobs [18], and this is stipulated to have been affected by all the factors that define "job satisfaction" [19]. These factors could be socio-demographic [20], personal feelings as in being able to self-express freely and be appreciated for work done [21], appropriate rewards, the number of hours of work, and promotion [22].

Job satisfaction among HCWs is said to be linked to some degree to the organization where they work as well as human relationships [16], both of which were earlier stated to significantly impact the quality of care provided and the general productivity [23,24]. Moreover, job satisfaction among HCWs, and the quality of care offered, is a determining factor for the success of an organization and the effectiveness of services provided in healthcare settings [25].

The satisfaction of HCWs at their jobs has received and continues to gain the attention of researchers in Saudi Arabia [26-28]. Reports in the Kingdom do not vary much with physicians and nurses who were indicated to show more satisfaction with their jobs than the paramedics, though differences were not significant [26]. The same report linked satisfaction at healthcare jobs to higher positions (physicians) as compared to those of HCWs holding lower positions. Also, the job satisfaction of HCWs was also linked to long years spent at the place of work [29]. However, according to documented evidence, overall job satisfaction among HCWs in Saudi has been reportedly low and this has led to turnover of workers [30].

With the Kingdom's rapid development, there is a shortage of HCWs, which is thought to be due to low levels of job satisfaction among caregivers [31]. This turnover of HCWs points to retention problems. In terms of retention of healthcare providers, it is of the view that retention of nurses in the Kingdom is a problem for hospitals as a result of low levels of job satisfaction [32]. However, the literature is silent on job retention due to non-reporting. Therefore, the need to investigate job satisfaction among frontline medical staff during the COVID-19 pandemic cannot be overemphasized, as this could serve as a point of reference in preparing for any future pandemics. This investigation was undertaken to this effect, taking into consideration the characteristic variables that had significantly influenced the factors affecting job satisfaction among HCWs. The study looks at job satisfaction among medical and allied health professionals in hospitals and PHCs during the COVID-19 pandemic in Riyadh, Saudi Arabia. It aims to compare responses from emergency responders and primary care providers, as well as any gender differences. A satisfied medical frontline professional means the retention of experienced staff who would most like to serve in cases such as public health emergencies in the future.

## Materials and methods

Study region, sample size, and ethical considerations

A cross-sectional descriptive investigation was used to evaluate job satisfaction among HCWs in emergency services and primary healthcare units in Riyadh, Saudi Arabia, for a period of six months from March 2020 to December 2021. The Riyadh region is geographically located in the centre of the Kingdom of Saudi Arabia and is reportedly the most populous city in the region as well as in the Kingdom (Saudi network: http://www.the-saudi.net/saudi-arabia/governorate.htm). An earlier report [31] specified that there were 42 hospitals and 363 PHCs in the region. Distributed among these were physicians (4924), pharmacists (440), personnel of allied health (5391), and technicians (1168). Thus, using Raosoft's [33] online sample size calculator, at a 5% margin of error and 50% response distribution, 375 physicians, 197 pharmacists, 359 allied health professionals, and 290 technicians would have been required to participate in the study at a 95% confidence level. However, to minimize the chances of error, a non-stratified sample size of 400 was taken due to the pandemic as there was a turnover in frontline HCWs. However, only 159 fully completed questionnaires were used for the investigation.

The research and its purpose were introduced to the participants. Participation in the survey was voluntary. Respondents' personal information was kept strictly confidential.

Ethical approval for the research was given by the IRB of Imam Mohammad Ibn Saud Islamic University, Riyadh, (approval number: 148-2021).

Research participants and collection of data

Medical staff who worked in the emergency department and PHCs during the COVID-19 pandemic between March 2020 to December 2021 were included. This included both genders of Saudi Arabian nationals and non-nationals. Members of the emergency department and primary healthcare medical staff who did not work during the pandemic period were excluded from the investigation. Data were collected using a self-administered, validated questionnaire consisting of both ordinal and nominal questions. Questionnaires were distributed online to healthcare clusters in the Riyadh region of Saudi Arabia. This consisted of governmental hospitals and PHCs. Questions were prepared in Google Forms (Google, Mountain View, CA), with the generated link distributed by email, online to professional websites and social media platforms, and by WhatsApp application. An expert team of researchers evaluated the content of the questionnaire for validity. Questions were also piloted with 10 HCWs, who confirmed their clarity and ease of following before being distributed. The 10 tested responses were excluded from the data.

The questionnaire comprised two sections, with the first section addressing demographical information such as gender, age, number of work hours per week, and number of years in practice. The second section was the job satisfaction survey (JSS), which encompassed 36 questions, divided into nine sub-dimensions with four items each on salary, promotion, fringe benefits, contingent rewards, supervision, operation procedures, co-workers, nature of work, and communication. The Likert five-point agreement scale was used in measuring the respondents’ levels of agreement and disagreement. The collected responses were reviewed, and consistency checks were carried out. Those that were incomplete with missing data were excluded from the analysis.

Statistical analysis

Data were collected on Excel sheets (Microsoft Corp., Redmond, WA) and analysed with SPSS version 23 (IBM Corp., Armonk, NY). GraphPad Prism version 9.2.0 (GraphPad Software, San Diego, CA) was used for heatmap analysis. Pearson's correlation coefficient was used to show the correlation between the proportion of disagreed and agreed responses and the significance was taken at p ≤ 0.05.

## Results

Demographic and job type descriptions of the respondents

**Figure 1:**
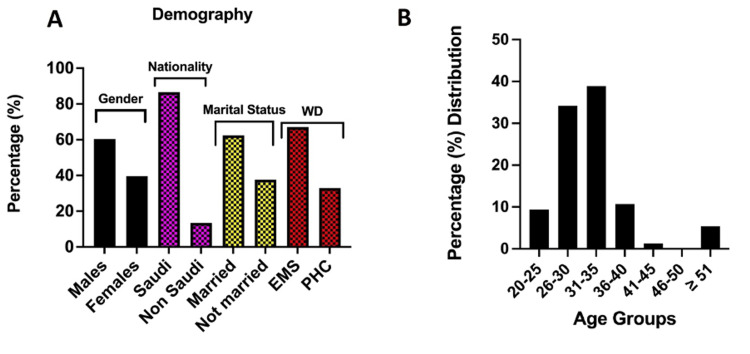
Demographic description of frontline medical healthcare workers during the COVID-19 pandemic. EMS: emergency medical services; PHC: primary healthcare centres.

A total of 400 professionals were targeted, and 159 HCWs responded to the questionnaire and were thus used for the investigation. Respondents included males (60.4%) and females (39.6%), the majority (86.6%) of whom were Saudi nationals. The distribution of demographic details is shown in Figures 1A, 1B. Results showed that 67.1% of the respondents were professionals working in EMS, while the remaining (32.9%) were PHC professionals. Married (62.4%) and unmarried (37.6%) people responded. The respondents' ages ranged from 20 to over 50 years (Figure 1B), with the majority being between 31-35 (38.9%) and 20-25 (34.2%) years old, respectively (Figure 1B).

**Figure 2:**
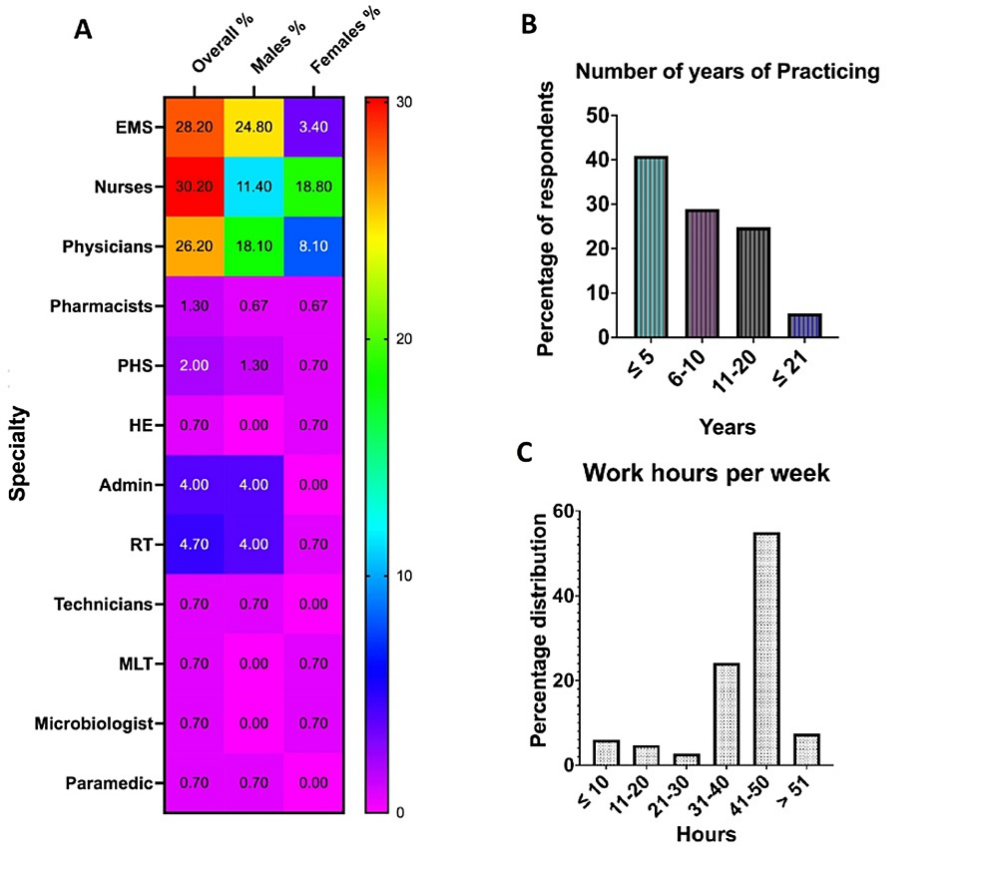
Heatmap showing gender distribution of profession/speciality (A), the years of practice (B), and working hours per week (C). The heatmap shows the overall specialities of the respondents and their distribution based on gender during the pandemic era. The colour bar indicates a percentage between 0% and 30%. EMS: emergency medical services; PHS: public health specialist; HE: health educationist; RT: respiratory therapist; admin: administration; MLT: medical laboratory technologist.

A heatmap of gender distribution based on profession/speciality is shown in Figure 2A. Nurses were the most prevalent (30.2%) among the respondents, followed by EMS professionals (28.2%) and physicians (26.2%). Respiratory therapists and administrative staff comprised 4.7% and 4.0%, respectively, of the respondents, while only 2% of them were PHC workers. The least number of respondents were healthcare educators, technicians, microbiologists, medical laboratory technologists, and paramedics, constituting 0.7% of each of the respondents (Figure 2A). The gender distribution as regards profession/speciality is also shown in Figure 2A.

The number of years of practice ranged from five to 20 years, and the distribution of hours worked as healthcare professionals ranged from 10 to 57 hours per week, with those working 41 to 50 hours per week having the maximum respondents (55%), followed by those working 31 to 40 hours per week, who constituted 24.2% of the respondents (Figures 2B, 2C).

Descriptive analysis of responses on rewards factors affecting job satisfaction among HCWs

In Table 1, responses to 12 questions aimed at evaluating the views of healthcare practitioners on rewards as they relate to job performance during COVID-19 are presented in percentages. The majority (56%) did not agree that they were paid a fair amount for the work, and the differences between them and those who affirmed by agreeing were not significant. Also, non-significantly more respondents (54.4%) expressed dissatisfaction with the benefits they received and significantly more (59%) agreed that raises were too few and far between. When benefits are compared with those of other organizations, 71.8% were of the view that they were not comparable with theirs (Table 1) while a large number (70.5%) disagreed that they get ahead fast in their places of work as compared to other organizations. Generally, the healthcare respondents found the job enjoyable but were not satisfied with the chances for promotion in their workplaces. They agreed rewards were few for them while disagreeing that benefit packages were equitable. There was an overall dissatisfaction reward that was received (Table 1).

**Table 1:** Responses on salary and rewards components of healthcare workers during the COVID-19 pandemic. The table shows the frequency of distribution of responses on the components of the questions on job satisfaction based on enumeration, reward packages, and promotion. Pearson's correlation coefficient was used to show the correlation between the proportion of disagreed and agreed. Significance was taken at p ≤ 0.05. It showed a strong negative correlation, which means that high disagreed response scores related to low agreed responses giving a value of R2, the coefficient of determination, as 1 with a p-value of 0.00001. The result is significant at p < 0.05.

Components of the questionnaire	Levels of disagreeing			Total % of disagree	Levels of agreeing			Total % of agree	
	Slightly	Moderate	Strongly		Slightly	Moderate	Strongly		
I feel I am being paid a fair amount for the work I do	10.1	17.4	28.9	56.4	22.1	10.1	11.4	43.6	
I am not satisfied with the benefits I receive	15.4	14.1	16.1	45.6	14.8	16.8	22.8	54.4	
Raises are too few and far between	17.4	10.8	12.8	41	18.1	16.1	24.8	59	
The benefits that I receive are as good as most other organizations	20.8	22.8	28.2	71.8	20.1	4.7	3.4	28.2	
I feel unappreciated by the organization when I think about what they pay me	18.8	15.4	16.2	50.4	16.8	15.4	17.4	49.6	
People get ahead as fast here as they do in other places	23.5	25.5	21.5	70.5	14.8	8.7	6	29.5	
The benefit package we have is equitable	23.5	22.1	22.9	68.5	17.4	6	8.1	31.5	
There are few rewards for those who work here	12.1	14.8	13.4	40.3	16.8	16.1	26.8	59.7	
I feel satisfied with my chances for salary increases	15.4	21.5	25.5	62.4	18.8	5.4	13.4	37.6	
There are benefits we do not have that we should have	6.7	12.1	13.4	32.2	13.4	14.8	39.6	67.8	
My job is enjoyable	17.4	10.7	12.8	40.9	16.8	18.1	23.2	58.1	
I am satisfied with my chances for promotion	18.1	16.8	26.2	61.1	20.8	10.7	7.4	38.9	

Descriptive analysis of responses of HCWs on work organization and supervisor roles

Job satisfaction responses to nine questions on the suitability of the work environment during the COVID-19 pandemic are shown in Table 2. There was an overall satisfaction shown by the respondents with the appropriateness of their work environment. Significantly more of the respondents (63.8%) agreed their supervisors were competent on the job and that supervisors were not unfair to them (69.8%), while also agreeing to the fact that they liked their supervisors (58.4%). In terms of work colleagues, a high percentage of respondents (71.8%) like those they work with as well as enjoy working with them (65.6%). A significantly higher percentage (62.5%) disagreed with too much squabbling and fighting at work, though the goals of the organization were not obvious to them.

**Table 2:** Analysis of the responses on work organization and supervisor roles by the healthcare workers during the COVID-19 pandemic. One sample t-test between proportions to determine whether differences between the percentage of those who agreed and disagreed were significant. T-statistic significance was taken at p ≤ 0.05. * = significant.

Components of the questionnaire	Levels of disagreeing			Total % of disagree	Levels of agreeing			Total % of agree	P-value
	Slightly	Moderate	Strongly		Slightly	Moderate	Strongly		
My supervisor is quite competent in their job	12.1	9.3	14.8	36.2	19.5	25.5	18.8	63.8	0.006*
I like the people I work with	11.4	9.4	7.4	28.2	14.1	30.2	27.5	71.8	0.00*
My supervisor is unfair to me	10.7	20.1	39	69.8	11.4	10.7	8.1	30.2	0.00*
I find I have to work harder at my job due to the incompetence of co-workers	19.5	22.8	15.5	57.8	18.1	13.4	10.7	42.2	0.05*
The goals of this organization are not clear to me	12.1	21.5	22.1	55.7	15.4	14.1	14.8	44.3	0.16*
My supervisor shows too little interest in the feelings of subordinates	18.8	20.1	16.9	55.8	18.1	10.7	15.4	44.2	0.15*
I enjoy with my co-workers	16.1	9.4	8.8	34.3	20.1	18.1	27.5	65.7	0.001*
I like my supervisor	13.4	14.8	13.4	41.6	16.8	23.5	18.1	58.4	0.03*
There is too much bickering and fighting at work	21.5	19.5	21.5	62.5	14.1	10.7	12.7	37.5	0.002*

Analysis of HCWs' responses to job satisfaction during the COVID-19 pandemic

Responses to the 15 questions by respondents on satisfaction with their job generally showed differences for 10 of them to be statistically significant, as shown in Table 3. This includes responses to chances of promotion on the job, which 58.4% agreed were too little, as well as the fact that they were not being recognized for a good job done (64.4%). The majority disagree that the work is meaningless (73.2%) as well as disagreeing (63%) that doing a good job could account for possible promotion. Most respondents agreed that they are appreciated for the work done (57.7%) while disagreeing with the fact that efforts to do a good job are seldom blocked by red tape (61%). Work done is appreciated (57.7%); however, there was an increase in workload (65.8%), but this had no effect on satisfaction as far as liking what they do at work (69.2%) and feeling proud of what they do (67.1%). On communication, 50.3% agree that they often do not know what is going on at their places of work, while 53% say yes, work assignments are fully explained, with differences not being significant (Table 3).

**Table 3:** Response analysis on job satisfaction by healthcare workers during the COVID-19 pandemic. One sample t-test between proportions to determine whether differences between the percentage of those who agreed and disagreed were significant. T-statistics significance was taken at p ≤ 0.05. * = significant.

Components of the questionnaire	Levels of disagreeing			Total % of disagree	Levels of agreeing			Total % of agree	P-value
	Slightly	Moderate	Strongly		Slightly	Moderate	Strongly		
There is really too little chance for promotion in my job	16.1	13.4	12.1	41.6	18.8	13.4	26.2	58.4	0.03*
When I do a good job, I receive the recognition for it that I should	18.1	18.8	27.5	64.4	16.8	12.1	6.7	35.6	0.003*
Many of our rules and procedures make doing a good job difficult	19.5	12.8	14	46.3	21.5	12.1	20.1	53.7	0.36
I sometimes feel my job is meaningless	16.8	24.2	32.2	73.2	11.4	9.4	6	26.8	0.00*
Communication seems good within this organization	16.1	19.5	14	49.6	22.8	14.8	12.8	50.4	0.92
Those who do well on the job stand a fair chance of being promoted	20.1	15.4	27.5	63	22.1	6.7	8.2	37	0.001*
I do not feel that the work I do is appreciated	20.1	20.1	17.5	57.7	20.8	8.7	12.8	42.3	0.05*
My efforts to do a good job are seldom blocked by red tape	22.8	22.1	16.1	61	18.8	12.8	7.4	39	0.006*
I like doing the things I do at work	11.4	12.1	7.3	30.8	16.8	21.5	30.9	69.2	0.000
I have too much to do at work	12.8	11.4	10	34.2	16.8	24.8	24.2	65.8	0.001*
I often feel that I do not know what is going on with the organization	18.1	16.8	14.8	49.7	20.1	13.4	16.8	50.3	0.94
I feel a sense of pride in doing my job	14.1	8.7	10.1	32.9	16.8	18.1	32.2	67.1	0.000*
I have too much paperwork	17.4	12.1	17.5	47	16.1	17.4	19.5	53	0.46
I don't feel my efforts are rewarded the way they should be	15.4	14.1	10.8	40.3	16.1	15.4	28.2	59.7	0.01*
The work assignment is not fully explained	18.1	18.1	16.8	53	20.1	12.1	14.8	47	0.46

Job satisfaction responses with respect to gender

Results on gender differences in HCWs' responses to being satisfied with their work during the COVID-19 pandemic are shown in Table 4. Gender-wise responses varied but were non-significant in most cases. More than half of the males (57%) and females (56%) disagreed with being fairly paid for work done. Also, 54% of both male and female respondents were not satisfied with the benefits offered. Additionally, both males (54%) and females (68%) agreed to the fact that raises were few, while more males (51%) did not feel unappreciated by their organization based on the pay received. However, differences based on gender were not significant (Table 4). There was general dissatisfaction with rewards, irrespective of gender. On the other hand, responses on the suitability of the work environment during the COVID-19 pandemic showed both males (63%) and females (64%) non-significantly agreeing to the competence of their supervisor as well as liking the people they worked with. There was a general satisfaction with the work environment, irrespective of gender. Thus, the frequencies of responses between male and female respondents, though varying, were not significant, as shown by the p-values in Table 4.

**Table 4:** Responses by gender on job satisfaction during the COVID-19 pandemic. Using a statistics calculator, a two-sample t-test between proportions of percentages was calculated to determine significant differences between male and female HCWs. * indicates significant differences between male and female responses to workload and rewards.

	Percentage (%)		P-value	Percentage (%)		P-value
	Disagree			Agree		
Job rewards	Males (N = 90)	Females (N = 59)		Males (N = 90)	Females (N = 59)	
I feel I am being paid a fair amount for the work I do	51 (57)	33 (56)	0.90	39 (43)	26 (44)	0.10
I am not satisfied with the benefits I receive	41 (46)	27 (46)	1.00	49 (54)	32 (54)	1.00
Raises are too few and far between	32 (36)	19 (32)	0.61	49 (54)	40 (68)	0.09
The benefits that I receive are as good as most other organizations	63 (70)	44 (75)	0.50	27 (30)	15 (25)	0.50
I feel unappreciated by the organization when I think about what they pay me	46 (51)	29 (49)	0.80	44 (49)	30 (51)	0.80
People get ahead as fast here as they do in other places	59 (66)	46 (78)	0.11	31 (34)	13 (22)	0.11
The benefit package we have is equitable	59 (67)	43 (73)	0.43	31 (34)	16 (27)	0.36
There are few rewards for those who work here	41 (46)	19 (32)	0.09	49 (54)	40 (58)	0.63
I feel satisfied with my chances for salary increases	52 (58)	41 (70)	0.09	38 (42)	18 (30)	0.14
There are benefits we do not have that we should have	33 (37)	15 (25)	0.12	57 (63)	44 (75)	0.12
My job is enjoyable	36 (40)	26 (29)	0.17	54 (60)	33 (56)	0.62
I am satisfied with my chances for promotion	51 (57)	40 (68)	0.17	39 (43)	19 (32)	0.17
Work environment						
My supervisor is quite competent in their job	33 (37)	21 (36)	0.90	57 (63)	38 (64)	0.90
I like the people I work with	21 (23)	21 (36)	0.70	69 (77)	38 (64)	0.08
My supervisor is unfair to me	62 (69)	42 (71)	0.79	28 (31)	17 (29)	0.79
I find I have to work harder at my job due to the incompetence of co-workers	53 (59)	33 (56)	0.71	37 (41)	26 (44)	0.71
The goals of this organization are not clear to me	55 (61)	28 (48)	0.12	35 (39)	31 (52)	0.12
My supervisor shows too little interest in the feelings of subordinates	52 (58)	31 (53)	0.54	38 (42)	28 (47)	0.54
I enjoy with my co-workers	34 (38)	17 (29)	0.26	56 (62)	42 (71)	0.26
I like my supervisor	35 (39)	27 (46)	0.39	55 (61)	32 (54)	0.39
There is too much bickering and fighting at work	54 (60)	39 (66)	0.46	36 (40)	20 (34)	0.46
Job satisfaction						
There is really too little chance for promotion in my job	39 (43)	23 (39)	0.62	51 (57)	36 (61)	0.62
When I do a good job, I receive the recognition for it that I should	57 (63)	39 (66)	0.73	33 (37)	20 (34)	0.73
Many of our rules and procedures make doing a good job difficult	45 (50)	24 (41)	0.28	45 (50)	35 (59)	0.28
I sometimes feel my job is meaningless	66 (73)	43 (73)	1.00	24 (27)	16 (27)	1.00
Communication seems good within this organization	42 (46)	33 (56)	0.23	49 (54)	26 (44)	0.23
Those who do well on the job stand a fair chance of being promoted	54 (60)	40 (68)	0.32	36 (40)	19 (32)	0.32
I do not feel that the work I do is appreciated	56 (62)	30 (51)	0.18	34 (38)	29 (49)	0.18
My efforts to do a good job are seldom blocked by red tape	53 (59)	38 (64)	0.54	37 (41)	21 (36)	0.54
I like doing the things I do at work	28 (31)	18 (31)	1.00	62 (69)	41 (69)	1.00
I have too much to do at work	35 (39)	16 (27)	0.13	55 (61)	43 (73)	0.13
I often feel that I do not know what is going on with the organization	48 (53)	26 (44)	0.28	42 (47)	33 (56)	0.28
I feel a sense of pride in doing my job	30 (33)	19 (32)	0.89	60 (67)	40 (68)	0.89
I have too much paperwork	48 (53)	22 (37)	0.05*	42 (47)	37 (41)	0.47
I don't feel my efforts are rewarded the way they should be	45 (50)	15 (25)	0.002*	45 (50)	44 (75)	0.002
The work assignment is not fully explained	52 (58)	27 (46)	0.15	38 (42)	32 (54)	0.15

Analysis comparing responses by work department

Results comparing responses by workers in emergency departments and primary health units are presented in Table 5. In terms of salary and benefits, HCWs in both healthcare settings were not satisfied, indicating that they were not adequately compensated during the period. Besides these, there were no increases in salary or rewards during this period. EMS and PHC professionals agreed that promotions were not rapid in their places of work when compared to other organizations. Hence, these professionals, irrespective of the work department, did not enjoy working during that period. Differences between EMS and PHC professionals on job satisfaction were not significant with p > 0.05 (Table 5). However, professionals from both work departments agreed that their supervisors were competent and that they liked the work environment. Also, they both agreed that their co-workers made the work easy. On overall job satisfaction, both EMS and PHC respondents agreed to not receiving recognition for their efforts during that time. During that period, they had to make do with a sense of pride in what they did even with much paperwork. In addition to this, they all agreed that rules and procedures made the job difficult (Table 5).

**Table 5:** Comparison of responses on job satisfaction by EMS and PHC workers during the COVID-19 pandemic. Using a statistics calculator, a two-sample t-test between proportions of percentages was calculated to determine the significant difference between EMS and PHC workers. * = significant. EMS: emergency medical services; PHC: primary healthcare centre.

	Percentage (%)		P-value	Percentage (%)		P-value
	Disagree			Agree		
Job rewards	EMS (N = 100)	PHC (N = 49)		EMS (N = 100)	PHC (N = 49)	
I feel I am being paid a fair amount for the work I do	60 (60)	24 (49)	0.20	40 (40)	25 (51)	0.20
I am not satisfied with the benefits I receive	37 (37)	17 (35)	0.80	56 (56)	31 (63)	0.41
Raises are too few and far between	46 (46)	16 (33)	0.13	54 (54)	33 (67)	0.13
The benefits that I receive are as good as most other organizations	73 (73)	34 (69)	0.61	27 (27)	15 (31)	0.61
I feel unappreciated by the organization when I think about what they pay me	46 (46)	29 (59)	0.13	54 (54)	20 (41)	0.13
People get ahead as fast here as they do in other places	65 (65)	40 (82)	0.03*	35 (35)	9 (18)	0.03*
The benefit package we have is equitable	67 (67)	35 (71)	0.62	33 (33)	14 (29)	0.62
There are few rewards for those who work here	39 (39)	21 (43)	0.64	61 (61)	28 (57)	0.64
I feel satisfied with my chances for salary increases	62 (62)	31 (63)	0.90	38 (38)	18 (37)	0.90
There are benefits we do not have that we should have	31 (31)	17 (35)	0.62	69 (69)	32 (65)	0.62
My job is enjoyable	42 (42)	19 (39)	0.72	58 (58)	30 (61)	0.72
I am satisfied with my chances for promotion	58 (58)	33 (67)	0.29	42 (42)	16 (33)	0.29
Work environment						
My supervisor is quite competent in their job	37 (37)	17 (35)	0.81	63 (63)	32 (65)	0.81
I like the people I work with	25 (25)	17 (35)	0.20	75 (75)	32 (65)	0.20
My supervisor is unfair to me	70 (70)	34 (69)	0.90	30 (30)	15 (31)	0.90
I find I have to work harder at my job due to the incompetence of co-workers	60 (60)	26 (53)	0.41	40 (40)	23 (47)	0.41
The goals of this organization are not clear to me	58 (58)	25 (51)	0.42	42 (42)	24 (49)	0.42
My supervisor shows too little interest in the feelings of subordinates	57 (57)	26 (53)	0.64	43 (43)	23 (47)	0.64
I enjoy with my co-workers	33 (33)	18 (37)	0.62	67 (67)	31 (63)	0.62
I like my supervisor	44 (44)	19 (39)	0.56	57 (57)	30 (61)	0.64
There is too much bickering and fighting at work	62 (62)	31 (63)	0.90	38 (38)	18 (37)	0.90
Job satisfaction						
There is really too little chance for promotion in my job	44 (44)	18 (37)	0.41	56 (56)	31 (63)	0.41
When I do a good job, I receive the recognition for it that I should	63 (63)	33 (67)	0.63	37 (37)	16 (33)	0.63
Many of our rules and procedures make doing a good job difficult	46 (63)	23 (47)	0.05*	54 (54)	26 (53)	0.90
I sometimes feel my job is meaningless	72 (72)	37 (76)	0.90	28 (28)	12 (24)	0.60
Communication seems good within this organization	51 (51)	23 (47)	0.64	49 (49)	26 (53)	0.64
Those who do well on the job stand a fair chance of being promoted	62 (62)	32 (65)	0.72	38 (38)	17 (35)	0.72
I do not feel that the work I do is appreciated	61 (61)	25 (51)	0.24	39 (39)	24 (49)	0.24
My efforts to do a good job are seldom blocked by red tape	59 (59)	32 (65)	0.48	41 (41)	17 (35)	0.48
I like doing the things I do at work	32 (32)	14 (29)	0.71	68 (68)	35 (71)	0.71
I have too much to do at work	34 (34)	17 (35)	0.90	66 (66)	32 (65)	0.90
I often feel that I do not know what is going on with the organization	45 (45)	29 (59)	0.11	55 (55)	20 (41)	0.11
I feel a sense of pride in doing my job	34 (34)	15 (31)	0.71	56 (56)	34 (69)	0.12
I have too much paperwork	49 (49)	21 (43)	0.49	51 (51)	28 (57)	0.49
I don't feel my efforts are rewarded the way they should be	39 (39)	21 (43)	0.64	61 (61)	28 (57)	0.64
The work assignment is not fully explained	50 (50)	29 (59)	0.30	50 (50)	20 (41)	0.30

## Discussion

The present study describes the responses of ED and primary healthcare professionals during the COVID-19 pandemic working as frontline HCWs. The finding of this study showed that they consisted more of males than females, the majority of whom were married. In some job satisfaction surveys, females are reported to have been more than males in the same region of this investigation [26,34]. It therefore showed that more male staff were used as frontline healthcare providers during the COVID-19 pandemic.

However, the non-significant differences between male and female responses as seen in the present study were similar to those of a recent report [14], but contrary to earlier reports by researchers in the pre-COVID-19 pandemic era [28,35].

Furthermore, our results showed that 64% of them were married, which is parallel to those of earlier reports [26,34]. It is therefore postulated that being married increased job satisfaction positively from an economic point [36]; however, our study also showed otherwise. Generally, the majority of the aspects that contribute significantly to being satisfied on a job were evaluated in this study. For example, respondents were significantly dissatisfied with the enumeration and rewards they received during the COVID-19 pandemic and this was irrespective of gender, and whether or not they were married. Dissatisfaction with rewards as seen here could simply mean that no new additional measures were put in place to reward these frontline medical and paramedical personnel during the COVID-19 pandemic. Consistent with the finding here on rewards during the COVID-19 pandemic are those of other reports [14,37]. Added to their frustrations is that possible promotion opportunities were not provided or put in place for these frontline HCWs.

It has been suggested that there is a need to negotiate or look into factors that affect job satisfaction generally as well as reinforcements in terms of salaries, rewards, bonuses, and promotions for HCWs, as they are perceived to be imperative for organizational growth and well-being [15]. Hence, in situations where there are no incentives offered to HCWs during the COVID-19 epidemic, they are likely to be less motivated [14] and this could be a contributing factor among the respondents in this survey. This might be an area that would need attention if healthcare workers are to be engaged in future frontline public health emergency services.

However, the work environment and working with colleagues were not issues here. The respondents were satisfied with their work environment and did get on well with those they worked with. The 71% of those who significantly indicated working harmoniously with their colleagues can be considered high, while the significant number of respondents who disagreed with squabbling and fighting at the workplace could possibly point to a cordial relationship among these COVID-19 pandemic frontline HCWs. This positive type of relationship and teamwork are needed in such a workplace, particularly during the pandemic and our findings here are similar to those of an earlier report [7]. There is the proposition that suggests frontline medical teams are temporary establishments, which are usually comprised of personnel with different specialities forming "comrades-in-arm" relationships to be able to fight and defeat the epidemic [7].

Another factor that can affect job satisfaction is appropriate leadership, which has been reported to be significantly correlated with job satisfaction [38]. In the present study, significantly more (69.8%) of the respondents were satisfied with their supervisors, thus suggesting appropriate leadership that generally affects job satisfaction, obligation, and suitable output by employees, as was earlier documented [39]. In addition, our findings showed that EMS and PHC professionals were highly satisfied with their supervisors, and this was irrespective of their gender, in accordance with an earlier report [28].

One other factor that has been shown to affect job satisfaction significantly is the speciality of the professionals [28]. This is because differences in job specialities present different work demands that create different variations in job stressors [39]. The results here revealed a high percentage of people (69.2%) who liked their jobs and did have a sense of pride in what they did, thus showing satisfaction in their career choice, similar to that reported by Abdulrahman et al. [27]. There is a possibility that due to the complexity of the COVID-19 epidemic and the difficult challenges of controlling and preventing its spread, frontline medical staff were assigned identical responsibilities that were not reflective of their degrees of specialization, as previously suggested [7]. This might explain why responses by both departments (EMS and PHC) here varied, but the differences were not significant. Therefore, variations in reports could be a result of burnout, stress, and the general framework of the pandemic.

Study limitations

One of the limitations of the study is the number of responses received for the investigation. Reminders were sent out to improve on this but did not advance the overall figure of respondents. Another limitation is the non-availability of data on the summation of frontline medical and allied health personnel in the region of this study during the period of the COVID-19 pandemic to help in calculating the expected sample size. The authors tried various recruitment approaches as described in the methodology in an attempt to improve the populace of respondents. Also, we failed to include a means of recording data of those who were frontline HCWs during the pandemic but who refused to participate. It is recommended to include information regarding the vaccination status of HCWs and the COVID-19 history of the participants.

## Conclusions

The present study has shown dissatisfaction among frontline HCWs during the COVID-19 pandemic. Some of the factors that led to job dissatisfaction were poor enumeration and rewards received during the period. In addition, there were no opportunities for promotion, which further heightened their dissatisfaction during the pandemic period. This in itself reflects some form of lack of appreciation for the risk they were exposed to in such a contagious pandemic. There is a need for stakeholders to monitor this aspect for improvement as qualified frontline HCWs might not be available in the future during any global health crises. However, satisfaction was expressed significantly in working with colleagues as well as pride shown in what they do as a profession. As expected, the workload was enormous, and opportunities for advancement were limited.

## Appendices

**Table 6:** Questionnaire.

Are you working in	Emergency department, Primary health care
Gender	Male, Female
Nationality	Saudi, Non-Saudi
Age	Open question
Marital status	Unmarried, Married
Profession/speciality	Physician, Nurse, EMS, Administrative, Other:……………….
How many years have you been practicing	Less than or equal to 5, 6-10, 11-20, More than 20
Work hours per week	Open question
Night duties per week	Open question
Daily sleep duration	Open question

36 questions	Disagree very much	Disagree moderately	Disagree slightly	Agree slightly	Agree moderately	Agree very much
I feel I am being paid a fair amount for the work I do.						
There is really too little chance for promotion in my job.						
My supervisor is quite competent in doing his/her job.						
I am not satisfied with the benefits I receive.						
When I do a good job, I receive the recognition for it that I should receive.						
Many of our rules and procedures make doing a good job difficult.						
I like the people I work with.						
I sometimes feel my job is meaningless.						
Communication seems good within this organization.						
Raises are too few and far between.						
Those who do well on the job stand a fair chance of being promoted.						
My supervisor is unfair to me.						
The benefits we receive are as good as most other organizations offer.						
I do not feel that the work I do is appreciated.						
My efforts to do a good job are seldom blocked by red tape.						
I find I have to work harder at my job because of the incompetence of people I work with.						
I like doing the things I do at work.						
The goals of this organization are not clear to me.						
I feel unappreciated by the organization when I think about what they pay me.						
People get ahead as fast here as they do in other places.						
My supervisor shows too little interest in the feelings of subordinates.						
The benefit package we have is equitable.						
There are few rewards for those who work here.						
I have too much to do at work.						
I enjoy with my co-workers.						
I often feel that I do not know what is going on with the organization.						
I feel a sense of pride in doing my job.						
I feel satisfied with my chances for salary increases.						
There are benefits we do not have that we should have.						
I like my supervisor.						
I have too much paperwork.						
I don't feel my efforts are rewarded the way they should be.						
I am satisfied with my chances for promotion.						
There is too much bickering and fighting at work.						
My job is enjoyable.						
Work assignments are not fully explained.						
